# Spheroid Culture of Mesenchymal Stem Cells

**DOI:** 10.1155/2016/9176357

**Published:** 2015-11-16

**Authors:** Zoe Cesarz, Kenichi Tamama

**Affiliations:** ^1^Department of Pathology, University of Pittsburgh School of Medicine, Pittsburgh, PA 15261, USA; ^2^McGowan Institute for Regenerative Medicine, University of Pittsburgh, Pittsburgh, PA 15219, USA

## Abstract

Compared with traditional 2D adherent cell culture, 3D spheroidal cell aggregates, or spheroids, are regarded as more physiological, and this technique has been exploited in the field of oncology, stem cell biology, and tissue engineering. Mesenchymal stem cells (MSCs) cultured in spheroids have enhanced anti-inflammatory, angiogenic, and tissue reparative/regenerative effects with improved cell survival after transplantation. Cytoskeletal reorganization and drastic changes in cell morphology in MSC spheroids indicate a major difference in mechanophysical properties compared with 2D culture. Enhanced multidifferentiation potential, upregulated expression of pluripotency marker genes, and delayed replicative senescence indicate enhanced stemness in MSC spheroids. Furthermore, spheroid formation causes drastic changes in the gene expression profile of MSC in microarray analyses. In spite of these significant changes, underlying molecular mechanisms and signaling pathways triggering and sustaining these changes are largely unknown.

## 1. Introduction

Multipotential stromal cells or mesenchymal stem cells (MSCs), originally isolated as single cell suspensions of bone marrow colonies of fibroblast-like cells adhering to plastic, carry multilineage differentiation potentials* in vitro* and* in vivo* after transplantation [1–6]. MSCs are relatively easy to obtain and to expand* in vitro* [7, 8].

Traditionally, two-dimensional (2D) adherent culture conditions have been used as a standard technique for* in vitro* expansion of MSCs. On the other hand,* in vitro* culture of multicellular aggregates was originally described for embryonic cells 70 years ago. Because of their spherical shape, these multicellular aggregates are now called multicellular spheroids, or spheroids. Spheroids have been utilized in the field of oncology [9, 10], stem cell biology [11–14], and tissue engineering [15, 16]. In this review, we will discuss an overview of spheroids and their significance in MSC biology.

## 2. Spheroids as Three-Dimensional (3D) Culture

2D cell culture is an easy and traditional culture condition; however, it is a highly artificial and less physiological environment, as some* in vivo* characteristics and traits are lost or compromised. In contrast, 3D cell culture is regarded as more physiological with these traits better preserved [10].

### 2.1. Spheroid Formation Techniques* In Vitro*

In the regular cell culture condition, anchorage dependent cells, including MSCs, in suspension will fall on the plastic surface by gravity and establish the cell adhesion to the plastic (strictly speaking, to the extracellular matrix (ECM) molecules such as fibronectin adsorbed on the plastic surface of cell culture plates and dishes via cell surface integrins) [17, 18]. In order to allow cells to form aggregates in suspension, these cells need to be cultured in a condition which does not allow them to adhere to a solid surface. Historically, the spinner flask method and the liquid overlay method had been used to facilitate cell aggregation [9]. The spinner flask method uses constant agitation of high density cell suspension to minimize cellular attachment to the solid surface and to maximize cell to cell contact, while the liquid overlay technique uses agar to prevent attachment. Early spinner flask and liquid overlay techniques result in a heterogeneous population of spheroids.

Later methods have improved upon the spinner flask and liquid overlay techniques to generate a more homogeneous population of spheroids. 96-well plates are now commercially available with low attachment surfaces for single spheroid production per well (e.g., 96 Well Ultra-Low Attachment Spheroid Plate from Corning in Corning, NY, or 3D-culture NanoCulture plate from Scivax in Tokyo, Japan); thus, spheroid size is determined by the number of cells in each well [19]. Another widely used technique for spheroid formation is the hanging drop method, which eliminates surface attachment by placing the cell suspension in a drop, allowing gravity to facilitate cellular aggregation at the bottom of the drop [20]. These cells spontaneously attach to each other to form cell aggregates if the possibility of surface attachment is abolished [20]. Another recent spheroid formation technique involves the use of chitosan membranes to initiate the 2D to 3D transition. Chitosan is a deacetylated derivative of a natural polysaccharide, chitin, and is often paired with another glycosaminoglycan, hyaluronan, known to have an impact on cell migration, proliferation, and matrix secretion [21, 22].

### 2.2. Spheroid Formation* In Vitro*

The formation process of multicellular spheroidal aggregates in low attachment conditions starts with the initial loose cell aggregate formation through integrin-ECM binding followed by the spheroid compaction through enhanced cell to cell connection via homophilic cadherin binding [15, 23, 24]. The formation of MSC spheroids was shown to be dependent on cadherins [25, 26] and the spheroid compaction was shown to rely on the actomyosin cytoskeleton [24]. Moreover, MSCs with intact endogenous ECM preserved by thermal lifting accelerate the initial cell aggregation process, as compared with trypsinized MSCs with degraded ECM [27]. Interestingly, the assembly process of MSC spheroids on the chitosan membrane is quite different from that in suspension or on nonadherent polymer surfaces. Rather than the self-aggregation present in other methods, MSCs attach and spread on chitosan membranes first and then retract their pseudopodia to form multicellular spheroids [21, 28].

### 2.3. Spheroid Culture in Oncology

Spheroidal cell culture has been used extensively in the field of oncology [9], as spheroidal cell culture exhibits both histological and physiological features similar to those of solid tumors in the body. Volume growth kinetics and spatial variation are better reproduced in 3D than in 2D culture [29–33]. Tumor spheroids synthesize ECM similar to original tumors* in vivo*, where the capacity for ECM production is reduced in the same cells in 2D culture conditions [34, 35]. The response of cancer cells to therapeutic interventions* in vivo* is better reproduced in* in vitro* spheroidal culture than in 2D adherent culture [29, 36–39]. In evaluating the efficacy of radiation therapy, spheroid culture of cancer cells produces a more comparable response to cells* in vivo* than cancer cells in 2D culture [9]. Additionally, tumor spheroids might possibly mimic circulating tumor cell aggregates [40–42].

### 2.4. Spheroid Culture in Stem Cell Biology

Spheroidal cell culture with pluripotent stem cells (PSCs), including embryonic stem cells (ESCs), is specifically called embryoid body [43–45]. Utilization of embryoid bodies is a standard protocol to produce specific cell lineages of interest* in vitro*, as the intercellular interactions of embryonic cells occurring during embryogenesis are recapitulated in the 3D culture setting [14]. Similarly, spheroidal cell culture of neural stem cells (NSCs), or neurospheres, has been used routinely for NSC isolation from embryonic and adult tissues and* in vitro* expansion and differentiation of NSCs into neurons, oligodendrocytes, and astrocytes [46, 47].

Differentiation capability and potential of stem and progenitor cells are generally enhanced in the 3D culture setting. For example, salivary gland-derived progenitor cells can differentiate into hepatocytic and pancreatic islet cell lineages, but these differentiations only take place when the cells are cultured in 3D cell aggregates, not in 2D monolayer [48]. Neuronal differentiation of ESCs is enhanced in embryoid body culture compared to 2D monolayer cell culture [49]. Moreover,* in vitro* reproduction of complex organ architecture, such as the optic cup, is made possible only in 3D culture, in which the inherent tissue self-organization capability of ESCs is maximized [11, 12].

### 2.5. Limitations in Spheroid Culture

There are some possible limitations known in the 3D spheroid culture technique. Because of the spheroidal structure, diffusion of nutrients, oxygen, and waste through the interior of the spheroids is compromised in a size-dependent manner [9, 10, 24]. Presence of these “stressors” can contribute to the characteristic gene expression profile of MSC spheroids; however, it can also compromise viability of the cells in the spheroid core, especially in harsh conditions [24] (see Section 3.4.5 and Section 4). Spinner flask techniques maximize the nutrient, oxygen, and waste diffusion through the spheroid, enabling larger spheroid culture and improving cell survival* in vitro* [9, 10, 24].

## 3. Significance of MSC Spheroids in Stem Cell Biology

### 3.1. Morphology and Mechanophysical Properties of MSC Spheroids

MSCs cultured in spheroids are spherical inside and elongated outside with an overall reduction of cytoskeletal molecules and ECM. The size of MSCs in spheroids is drastically smaller than cells in 2D monolayer, resulting in 75% reduction in individual cell volume [24, 50–52]. Cellular morphology is a key characteristic used to determine cellular phenotypes and fates of MSCs [53]. Small, rounded MSCs are prone to differentiate into an adipogenic lineage, whereas large, extended MSCs are prone to differentiate into an osteogenic lineage in both 2D and 3D culture system [54, 55]. Moreover, these differentiation preferences in MSC spheroids can be altered by myosin II inhibitor blebbistatin or constitutively active Rho kinase treatments, indicating the pivotal role of actomyosin cytoskeleton and myosin-generated mechanical tension in these processes [55].

Another major difference between 2D monolayer culture and 3D spheroid culture is Young's elasticity modulus of the materials surrounding the cells, which should also affect cell differentiation [56, 57]. The cells in 2D regular monolayer reside on plastic with an elasticity modulus in the gigapascal (GPa) range, whereas cells in 3D spheroids should be surrounded by the cells and ECM with a combined elasticity modulus of less than 0.1 kPa [52]. The biological significance of the elasticity modulus has only been addressed in 2D monolayer culture [56, 57], and it should also contribute to the altered gene expression and cell phenotype in 3D spheroids. All of these data indicate the clear difference in mechanophysical properties between spheroidal MSCs and MSCs in 2D monolayer culture on plastic [13].

### 3.2. Gene Expression Changes in MSC Spheroids

Microarray analysis showed a drastic change in the gene expression profile in the MSC spheroid culture when compared with MSCs in 2D monolayer culture with upregulation of 1,731 genes and downregulation of 1,387 genes [58]. The upregulated genes are associated with hypoxia, angiogenesis, inflammation, stress response, and redox signaling, including angiopoietin 2 (*ANGPT2*), bone morphogenetic protein 2 (*BMP2*), chemokine (C-X-C motif) receptor 4 (*CXCR4*), heme oxygenase 1 (*HMOX1*), interleukin 1*α* (*IL1A*), interleukin 1*β* (*IL1B*), interleukin 6 (*IL6*), interleukin 8 (*IL8*), interleukin 11 (*IL11*), interleukin 24 (*IL24*), leukemia inhibitory factor (*LIF*), prostaglandin-endoperoxide synthase 2/cyclooxygenase 2 (*PTGS2/COX2*), tumor necrosis factor *α*-induced protein 6/tumor necrosis factor *α* stimulated gene/protein 6 (*TNFAIP6/TSG6*), transforming growth factor-*β*3 (*TGFB3*), and vascular endothelial growth factor-A (*VEGFA*) [28, 50, 58–60]. Moreover, stronger induction of gene expression of key genes of interest, such as* BMP2*,* LIF*,* PTGS2/COX2*, and* TGFB3*, is observed in MSC spheroids formed on chitosan membranes than the ones formed on a nonadherent surface [28]. The molecular mechanisms responsible for the altered gene expression profiles in MSC spheroids are largely unknown (see below).

Quantitative reverse transcription- (qRT-) PCR is the major method for quantitative analysis of targeted gene expression in current cell biology research. As discussed earlier, gene expression of cytoskeletal molecules including *β*-actin (*ACTB*) is largely reduced in MSC spheroids [28, 50].* ACTB* is frequently used as an endogenous normalizer in gene expression analysis; therefore, utilization of* ACTB* as an endogenous normalizer could lead to possible overinterpretation of upregulated genes in 3D MSC spheroids and thus data analysis and interpretation of gene expression need caution.

### 3.3. *In Vivo* Counterpart of MSC Spheroids

As discussed earlier, tumor spheroids are an* in vitro* imitation of the original tumors* in vivo*, whereas embryoid bodies are an* in vitro* imitation of the inner cell mass in blastocysts. However, an* in vivo* counterpart of MSC spheroids is not immediately clear. Intravenously administered single cell suspension MSCs form cell aggregates and are trapped as emboli in lung. These cells could possibly cause harmful effects to the recipients through MSC-derived pulmonary emboli, especially if a massive dose of MSCs is transplanted intravenously [61], but at the same time these cells also express* TNFAIP6/TSG6* very strongly, similar to MSC spheroids, exerting strong anti-inflammatory effects [50, 62]. Endogenous MSCs reside as a subfraction of pericytes surrounding the vasculature [63–68]. Pericytes in noninjured tissues are not activated, whereas cultured MSCs are counterparts of activated pericytes found in repairing and regenerating tissues, such as granulation tissues [69]. Granulation tissues are comprised of loose cellular aggregates, including pericytes embedded within provisional ECM, although compact spheroidal cell aggregates are not typically observed in granulation tissues [70].

### 3.4. Clinical Significance

**Figure 1:**
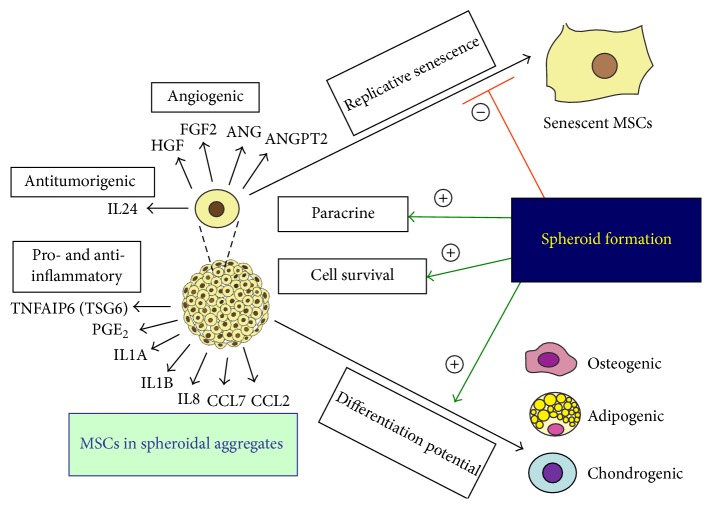
Clinical significance of MSC spheroids. Formation of spheroidal aggregates (1) enhances paracrine secretion of angiogenic, antitumorigenic, and pro- and anti-inflammatory factors, (2) improves cell survival, (3) increases differentiation potentials, and (4) delays replicative senescence of MSCs (ANG: angiogenin; ANGPT2: angiopoietin 2; CCL2: chemokine (C-C motif) ligand 2; CCL7: chemokine (C-C motif) ligand 7; FGF2: fibroblast growth factor 2; HGF: hepatocyte growth factor; IL1A: interleukin 1*α*; IL1B: interleukin 1*β*; IL8: interleukin 8; IL24: interleukin 24; PGE_2_: prostaglandin E_2_; TNFAIP6 (TSG6): tumor necrosis factor *α*-induced protein 6 (tumor necrosis factor *α* stimulated gene/protein 6); VEGFA: vascular endothelial growth factor-A).

MSC-based therapeutics is a promising approach in the field of autoimmune diseases, regenerative medicine, and tissue engineering. However, the beneficial effects of MSC-based therapeutics in initial small scale clinical studies are often not substantiated by large randomized-controlled clinical trials, strongly indicating the urgent need of further optimization of cell-based therapy [71–73]. There are various approaches to improve the efficacy of MSC-based therapeutics, and MSC preparation as spheroids represents one method of optimization. Spheroid formation has been shown to enhance anti-inflammatory effects, augment tissue regenerative and reparative effects with enhanced angiogenesis, facilitate differentiation potentials of multiple lineages, increase posttransplant survival of MSCs, improve MSC stemness, and delay* in vitro* replicative senescent processes, as discussed in detail below (Figure 1).

#### 3.4.1. Enhanced Anti-Inflammatory Effects

MSCs exert strong anti-inflammatory or immunomodulatory effects and MSC-based therapeutics is regarded as promising approaches against immune-mediated diseases, such as graft versus host disease. Multiple molecules, such as indoleamine dioxygenase-1 (IDO1) or prostaglandin E_2_ (PGE_2_), have been identified to mediate MSCs' strong anti-inflammatory effects [71, 74–77]. Recently, MSC spheroids were shown to exert strong anti-inflammatory effects, presumably through upregulated TNFAIP6/TSG6 produced by MSC spheroids [50]. In this study, MSC spheroids reduced macrophage activation in a coculture system* in vitro* or mitigated zymosan-induced inflammation in a mouse zymosan-induced peritonitis model. PGE_2_ is another molecule strongly upregulated in MSC spheroids [78].

Interestingly, strong upregulation of these mediators in MSC spheroids is observed when MSC spheroids are cultured in the regular cell culture medium (i.e., alpha MEM supplemented with FBS), but this upregulation is largely abolished if the MSC spheroids are cultured in the serum and animal component-free chemically defined medium (MesenCult-XF Medium, STEMCELL Technologies, Vancouver, Canada) [19]. The apparent reason for this difference is unknown, but this result clearly indicates the presence of unknown factors in serum pivotal for the upregulation of immunomodulatory mediators in MSC spheroids. Xeno-free chemically defined media are ideal for* in vitro* preparation of clinical-grade MSCs [8], but anti-inflammatory or immunomodulatory effects might not be reproduced in MSCs cultured in chemically defined media [19, 79], indicating the importance in learning the underlying molecular mechanisms of MSCs' strong immunoregulatory properties.

As seen above, spheroidal formation upregulates proinflammatory cytokines (such as IL1A, IL1B, and IL8) and chemokines (such as chemokine (C-C motif) ligand 2 (CCL2) and chemokine (C-C motif) ligand 7 (CCL7)) that recruit inflammatory cells, indicating possible proinflammatory properties of MSCs [23, 28, 50, 58]. MSCs are required to be primed with proinflammatory cytokines to acquire anti-inflammatory properties [71, 74, 80], and MSCs in spheroids are self-stimulated by autocrined IL1 signaling to have enhanced anti-inflammatory effects [23]. In other words, MSC spheroids use autocrined proinflammatory cytokines as molecular switches of their anti-inflammatory properties. But at the same time it is also possible that these proinflammatory cytokines produced by MSC spheroids directly contribute to the inflammatory response of the host, though this possibility has not been shown experimentally. Moreover, recent studies have shown that MSCs promote recruitment of inflammatory cells. This can be interpreted as proinflammatory effects of MSCs [81–83], but it is also shown to be required for MSC-mediated anti-inflammatory effects [84]. Thus, underlying molecular mechanisms of MSC-mediated anti-inflammatory and possibly proinflammatory effects are very complicated. Further studies are required to address the possible proinflammatory roles of these proinflammatory cytokines and chemokines strongly produced by MSC spheroids, as these proinflammatory cytokines or chemokines could directly enhance inflammation around the MSC spheroids in certain situations.

#### 3.4.2. Enhanced Angiogenic and Tissue Reparative/Regenerative Effects

Tissue repair and regeneration are an essential biological function for humans. In this complex biological process, numerous types of cells and bioactive mediators are regulated in a temporary and spatially sophisticated manner. The normal repair process of adult tissues, represented with skin in this case, takes place in three phases: inflammation, new tissue formation, and remodeling.* Inflammation* is an initial body's adaptive response to tissue damage, comprised of hemostasis and recruitment of inflammatory cells. The* new tissue formation* phase involves cellular proliferation and migration of various cells, such as endothelial cells or fibroblasts, and ECM production by these cells to form granulation tissues. New blood vessel formation or angiogenesis provides conduits of cellular and nutritional supports to the granulation tissues. The* remodeling* phase involves termination of the active repair process, reduction of these cells by emigration or apoptosis, and wound contraction by myofibroblasts to leave fibrous scar tissues consisting of disorganized ECM deposits in the end [85]. Contrary to adult wound healing, scar formation does not happen in mammalian early fetuses (before day 16 of mice), which retain tissue regenerative capacity. The major difference in the tissue repair process between fetuses and adults lies in the inflammation phase, which does not take place in the tissue repair of early fetuses. Consistently, scar formation is reduced by inhibiting inflammation [86].

Gene expression of various growth factors and cytokines, including angiogenin (*ANG*),* ANGPT2*, fibroblast growth factor 2 (*FGF2*), hepatocyte growth factor (*HGF*), and* VEGFA*, is upregulated in MSC spheroids [28, 58, 59]. ANGPT2 activates endothelial cells and exerts a strong angiogenic response in the presence of VEGFA [87, 88]. FGF2 and HGF are also angiogenic molecules [89–91]. Thus, it is logical to speculate that MSC spheroids are more tissue reparative through their stronger angiogenic effects than MSCs cultured in monolayer, and it was indeed shown in various animal models [26, 51, 92–96].

HGF-mediated antifibrotic effects have been reported for MSCs [97]. Moreover, TGFB3 has been shown to be a key mediator for scar-free skin repair or skin regeneration [86]. Both HGF and TGFB3 are upregulated in MSC spheroids [28] and thus MSC spheroids might have stronger antifibrotic or even tissue regenerative effects than MSCs cultured in monolayer.

#### 3.4.3. Enhanced Stemness and Delayed Replicative Senescence

A key feature of MSCs is their multilineage differentiation potentials, which have drawn attention in the field of regenerative medicine [1–6]. Initially, MSC spheroids or pellets were solely utilized for their chondrogenic differentiation capacity [98–100]. It was subsequently realized that differentiation potentials of MSC spheroids are enhanced not only to the chondrogenic lineage, but also to other lineages [21, 28, 60, 101–104].

Another interesting feature of MSC spheroids is that spheroidal formation prolongs replicative lifespan or delays cell senescence of MSCs* in vitro* [95]. This study also shows the increased gene expression of pluripotency marker genes (*NANOG*,* SOX2*, and* POU5F1/OCT4*) in MSC spheroids, consistent with previous studies [21, 102]. However, the degree of pluripotency gene upregulation is relatively weak in these studies and the role of OCT4 in adult stem cells has been questioned [105, 106]. Thus, interpretation of the roles of upregulated pluripotency marker genes in MSC spheroids needs caution.

Enhanced multilineage differentiation potentials, delayed cell senescent processes, and upregulation of pluripotency marker genes are indicative of enhanced stemness in MSC spheroids. This concept is supported by colony formation assays, which measure the proportion of early progenitors in culture [107]. Colony formation capability is increased with MSC suspension derived from spheroids as compared with that from MSCs cultured in monolayer, further indicating enhanced stemness in MSC spheroids [108]. MSC spheroids derived from thermally lifted cells have enhanced differentiation and colony formation potential, as compared with MSC spheroids from trypsinized MSCs, indicating the pivotal role of intact ECM for stemness preservation of MSC spheroids [27].

Although ease of* in vitro* preparation is a great strength of MSC-based therapeutics [5, 7, 8, 109],* in vitro* MSC expansion leads to replicative cell senescence, loss of differentiation potentials, and reduced paracrine capability so that organ protective effects become compromised [110–115].* In vitro* preservation of MSC stemness is one of the clinical significant aspects of MSC spheroids.

#### 3.4.4. Possible Enhancement of Antitumorigenic Effects

IL24 is a multifunctional cancer killing cytokine [116–118] that is a strongly upregulated gene in MSC spheroids [28, 50, 60]. Interestingly, MSC spheroids were shown to selectively reduce the viability of cancer cell lines but not that of noncancer-derived immortalized cell lines in an IL24-dependent mechanism, suggesting that MSC spheroids might be utilized in novel cancer therapeutics [60]. As seen earlier, MSC spheroids have enhanced production of growth factors and cytokines including mitogens [28, 50, 58–60]. Indeed MSCs have been shown to have protumorigenic effects by secreting growth factors and cytokines and directly contributing to tumor stroma, in addition to their antitumorigenic effects [119]. Moreover, transformation of MSCs themselves is another potential concern [120]. The concern might be more legitimate with MSC spheroids, as stemness of these cells is enhanced (see above). Overall, it appears that pro- or antitumorigenic effects of MSCs are largely context-dependent. Thus, it is very attractive to hypothesize MSC spheroids as novel therapeutics for certain cancers, but more rigorous studies are needed to address this hypothesis.

#### 3.4.5. Improved Cell Survival after Transplantation

One of the factors limiting the efficacies of MSC therapeutics is posttransplant cell survival [8, 121]. An early study showed that >99% of MSCs transplanted to the uninjured heart are cleared within 4 days after cell injection [122], whereas another study showed that >85% of systematically injected MSCs are entrapped and lost in precapillaries [123]. Even though MSCs exert tissue reparative and regenerative effects presumably through a brief “hit and run” mechanism and thus long-term engraftment might not be a prerequisite for the tissue reparative and regenerative effects of MSCs [76, 124], initial survival of transplanted MSCs should nevertheless be a critical factor defining the overall efficacy of MSC-based therapeutics. MSC spheroids have been shown to have improved survival* in vivo* compared to single cell suspensions of MSCs [93], even though MSC spheroids are shown to have less survival advantage than MSCs in 2D regular condition* in vitro* [24] (see Section 2.5 and Section 4). Additionally, the antiapoptotic molecule Bcl-2 is upregulated while the proapoptotic molecule, Bax, is downregulated resulting in an overall prosurvival molecular profile in spheroidal cells [93]. Improved survival of posttransplanted MSCs contributes to the enhanced therapeutic efficacy of MSC spheroids* in vivo*.

## 4. Key Molecular Signals and Events in MSC Spheroids

Despite the promising potential that MSC spheroids have in regenerative medicine and autoimmune diseases, there is limited research on the underlying molecular mechanisms and signaling pathways which initiate and mediate these drastic differences in the gene expression profile and phenotype of MSC spheroids.

Oxygen reaches the inside of spheroids through diffusion, which makes the internal core of spheroids hypoxic [9, 13, 15]. Consistently, hypoxia-associated genes, such as* VEGFA*, are overrepresented among the upregulated genes in MSC spheroids in the microarray analysis [28, 58]. Hypoxia inducible factor (HIF) is a master transcription factor that regulates expression of hypoxia-associated genes [125]. MSCs express HIF-2*α* in addition to ubiquitous HIF-1*α* [126], and we showed that HIF-1*α* and HIF-2*α* have a limited but important role in MSC self-renewal and production of growth factors and cytokines in hypoxia [127]. HIF-2*α* is also identified as one of the stemness genes in human MSCs [128]. Protein expression of both HIF-1*α* and HIF-2*α* is observed in MSC spheroids [51], and, thus, both HIF-1*α* and HIF-2*α* should serve as key transcription factors in MSC spheroids.

The self-aggregation process of MSCs initiates caspase-dependent IL1 autocrine signaling. We have previously shown that one of the signaling molecules upregulated by IL1 is early growth response gene-2 (EGR2), a zinc finger transcription factor that regulates PGE_2_ levels through regulation of* PTGS2/COX2* gene expression in MSCs [7, 129].* EGR2* expression is upregulated in MSC spheroids [28], presumably in response to autocrined IL1 stimulation [7, 129], and the enhanced anti-inflammatory properties of MSC spheroids should be attributable to upregulated* EGR2*, at least partly.

The IL1 autocrine signaling subsequently upregulates chemokine receptors, such as CXCR4, or immunomodulatory mediators, such as TNFAIP6/TSG6, IL6, and PGE_2_ [23, 24]. Interestingly, spheroidal formation coincides with reduced mitochondrial membrane potential and ATP production, indicating the ongoing apoptosis process in MSCs in spheroidal aggregates [24]. Apoptotic cells are shown to process and release IL1 [130]. Furthermore, MSCs in spheroidal aggregates are shown to have higher fluorescent calcium uptake than MSCs in 2D culture [25], and intracellular calcium overload is regarded as apoptogenic [131–133]. Thus, the apoptotic process seems to trigger the IL1 autocrine signaling and induce the stress response in MSC spheroids [24]. However, it cannot reconcile well with their improved cell survival* in vivo* [93]. One possible explanation is quick disassembly of MSC spheroids after transplantation eliminating compromised oxygen and nutrient access to the interior of spheroids as a factor (see Section 2.5). In fact, disassembled MSCs from MSC spheroids have a survival advantage over MSCs cultured on regular 2D condition* in vitro*, supporting such an explanation [24, 95].

Even though a glimpse of key signaling pathways has been revealed, more studies of crucial molecular events and signaling need to be conducted. For example, the signaling pathways connecting the initial self-aggregation process and the apoptotic process are still unknown. Moreover, the upstream signaling events causing such a drastic change in the gene expression profiles in MSC spheroids [28, 50, 58–60] are largely unclear. As discussed earlier, the alteration in mechanophysical properties might be such a significant event in MSC spheroids [13], but it requires experimental validation.

Epigenetics is defined as an inheritable change in gene expression through DNA methylation, noncoding RNAs, and histone modification, without altering the DNA sequence itself [134–136]. It causes drastic changes in gene expression profiles, as best exemplified in the fertilization process and the somatic cell reprogramming process during the development of induced pluripotent stem cells (iPSCs) [137]. Indeed, MSC spheroids were shown to acquire epigenetic changes. In this study, histone H3 lysine 9 acetylation (H3K9ac), which favors transcriptional activation [138], increases in promoter regions of* NANOG*,* SOX2*, and* POU5F1/OCT4 *and telomerase reverse transcriptase (*TERT*) in MSC spheroids, as compared with MSCs cultured in monolayer. Thus, epigenetic regulation appears to be one of the underlying molecular mechanisms causing the drastic change in the gene expression profile in MSC spheroids.

## 5. Epilogue

MSCs have shown promise in the field of regenerative medicine and 3D MSC culture further enhances such characteristics. Microarray analysis has shown a drastic change in the gene expression profile between monolayer and spheroid cultured MSCs; however, a critical lack of understanding exists with relation to the molecular signaling mediating the enhanced MSC spheroid properties or the improved cell survival. More mechanistic work is definitely needed at the molecular level to better understand and optimize MSC spheroids for clinical applications.
